# Supplementary material for: Are carbon emissions trading and green financial instruments synergistic? -Comprehensive quantitative research based on content analysis
Source: PLoS One. 2024 Mar 7;19(3):e0298601. doi: 10.1371/journal.pone.0298601 (PMC10919601; doi:10.1371/journal.pone.0298601)
Supplement: S1 Data — (ZIP) [file pone.0298601.s001.zip › Data/地级市Z_F工作报告.zip!/╜¡╦╒/╦▐╟¿╩╨/└┤╫╘╣1⁄2╓┌║┼í╛┬φ┐╦╩2╛▌═°í┐_2017.docx]

各位代表：

现在，我代表市人民政府向大会作工作报告，请予审议，并请市政协各位委员和其他列席同志提出意见。

过去五年政府工作回顾

本届政府履职的五年，是宏观形势变化最大、压力挑战最多的五年，也是宿迁建市20年来最不平凡的五年。五年来，我们在省委、省政府和市委的坚强领导下，在市人大、市政协监督支持下，深入贯彻党的十八大、十八届三中四中五中六中全会和习近平总书记系列重要讲话精神，紧紧围绕全面建成小康社会的奋斗目标，自觉践行“五大发展理念”，突出“三抓三促”工作主线，扎实做好稳增长、促改革、调结构、惠民生、防风险各项工作，经济社会发展呈现出“总体平稳、稳中有进、进中向好、结构优化、质量提升”的良好态势，完成了市四届人大各次会议确定的目标任务，开创了宿迁改革发展稳定的新局面。

——一批综合性指标增幅持续领先，实现经济总量跻身全国百强的历史性跨越。坚持超前研判、精准施策、积极作为，有效顶住了经济下行压力，经济发展始终保持快于全省、好于周边的势头。地区生产总值突破2000亿元大关，由1522亿元增至2351亿元，成功跻身全国地级市百强，年均增长10.6%，分别快于全国、全省3.4个、2个百分点；人均GDP从5042美元增至7276美元，达到中上等收入国家水平；一般公共预算收入突破200亿元，由158.1亿元增至238.1亿元、位列全国70强，年均增长10.8%；列统的16项主要经济指标中有10项年均增幅达两位数，5项指标增幅位居全省第一，成为江苏乃至长三角地区发展速度最快、综合实力提升最明显的地级市之一。

——一批质量性指标持续向好，经济社会发展活力日益增强。长期以来，宿迁经济发展高度依赖房地产业，自2014年起停止房地产开发用地出让挂牌，经济并没有出现大幅下滑，主要源于工业经济的提质增效。工业用电量跃居苏北中游，在全力推动钢铁行业去产能的背景下，去年全市工业用电量增速仍然达到9.2%，年均增幅达9.7%，均居全省第一，总量由全省末位攀升至第十一位；工业增值税保持快速增长，2016年增速达23.8%、年均达15.6%，均居全省第一；规上企业主营业务收入利润率连续五年全省第一，年均达10%、高于全省平均3个百分点。工业制造业的加快发展，带动了更多群众就业，老百姓的口袋更加殷实，全市私人汽车保有量升至苏北第三、达44.85万辆，较2012年翻了一番多；经济发展活跃度越来越高，各方面主体活力竞相迸发，从移动电话、移动互联网、互联网宽带用户数看，总量均实现翻番，分别达392.5万户、339万户、88.9万户，均由全省末位上升至第十一位。这些一个个量的积累，正加快汇聚成宿迁实现转型发展、跨越赶超的强大动力。

——一批国字号荣誉成功获批，以崭新的形象确立了宿迁在新兴城市中的重要位置。坚持以民意为基础，以需求为导向，以“五城同创”为抓手，补欠账、强功能、塑精品，个性特色日趋增强，文明程度大幅提升，城市形象和功能发生根本性改变，成功摘得中国人居环境奖城市、国家卫生城市、国家节水型城市、全国双拥模范城、中国人居环境范例奖、省优秀管理城市等一大批桂冠。发展的活力和潜力被国际知名智库充分认可，宿迁对外“落后”的印象得到有效扭转，赢得了各方赞誉，广大市民的认同感和自豪感不断增强。

——一批原创性改革率先推出，为全国改革作出了积极贡献。坚持以激活经济社会发展活力为目标，在全国率先推出资格资质去行政化、“一证一号”、工业类产品生产许可制度、国家扶贫改革试验区“四项机制”等一批原创性改革，形成了全面深化改革的“宿迁经验”，大大降低了市场和社会主体的制度性交易成本，一般性企业设立等待时间由过去1个多月压缩至3个工作日内，政府类投资建设项目综合纯审批时限由102个工作日压缩至40个工作日，非政府类投资项目综合纯审批时限由92个工作日压缩至32个工作日。我市改革对全国面上改革起到了积极的推动作用，得到各方高度关注，央视等主流媒体多次聚焦报道，中央和省领导给予充分肯定，宿迁改革的金字招牌进一步擦亮。

——一批“老大难”问题有效解决，群众的期盼和诉求得到实实在在的回应。始终围绕群众反映强烈、意见最多的问题开展工作。下决心推进马陵河综合整治，原先的“龙须沟”已发生巨大变化。根据国家和省统一部署，全面取缔骆马湖非法采砂，十多年不见的银鱼鱼汛得以重现。按照“彻底禁、禁彻底”的要求，全面完成耿车、三棵树等重点区域废旧物资综合整治，全市6978户废旧物资非法回收加工户全部整改，清理废旧物资60余万吨，清运填埋垃圾14.4万吨，整治河塘沟渠600余个，栽植苗木100余万株，一举解决了困扰群众20多年的环境污染问题，基本实现了生产转型、生活富裕、生态美好的整治目标。立足“疏堵结合”，把严格管理和最大程度照顾从业人员利益结合起来，实现市区“大花轿”全面禁行。全方位整治户外广告，市区楼顶和跨街广告牌彻底“下岗”，城市形象大幅提升。大力实施区域供水“通村达户”工程，总投资达45.7亿元，是建市以来投资额最大、惠及面最广的民生工程；累计铺设各类管网5万千米，区域供水通村率达100%，达户率超过90%，农村居民用上与城市居民同源同网同质的自来水。推动市区水价大幅下调，居民、企业用水价格分别由全省第二位、第五位下降至第十位、第十三位，群众和企业获得感进一步增强。

五年来，我们持之以恒抓项目、促转型，产业质态明显提升。把做大做强产业作为第一要务，突出集聚升级，优化产业布局，强化产业培育，产业发展实现量质齐升。五大任务扎实推进。以落实“三去一降一补”为抓手，积极推动产业转型升级。坚决化解钢铁等过剩产能，顺利完成省定目标任务；出台“房六条”政策，积极化解房地产库存，市区商品住宅去化周期由最高的28个月缩短至11.5个月；严控政府债务规模，降低企业融资成本，直接融资占比提升12.34个百分点、达21.4%；持续压减收费项目、降低收费标准，工业用气用水价格降至全省最低；聚焦重点产业、重大基础设施、民计民生等领域，深入开展“找补强”活动，发展的整体性和协调性进一步增强。工业质态不断优化。围绕市域“4+4”、县区“2+1”产业定位，扎实推进“3+3”基地建设，大力实施“511”工程，工业总产值达4313亿元、年均增长19.9%，工业增加值达1090亿元、年均增长13.8%，增速均居全省第一。工业经济总体呈现“五升三降”的良好态势：“五升”，即特色产业优势持续提升，食品饮料、纺织服装、机电装备、家居制造四大特色产业销售均突破500亿元，总产值翻了近一番、达2500亿元，特色产业优势更加明显。新兴产业实力持续提升，智能家电、绿色建材、功能材料、智能电网四大新兴产业总产值达448亿元、占比达10.4%。智能家电产业引进18个重大项目，冰箱、制冷压缩机、智能液晶电视等成功下线，改变了我市长期以食品、板材等为主的传统产品结构；中国绿色建材产业合作论坛永久落户宿迁，康美新材料、大亚圣象地板等一批重特大项目顺利落户，绿色建材产业产值突破100亿元、年均增长18.6%，智能家电、绿色建材双双获批省级先进制造业基地，顺利通过中国家电产业基地验收评审。高新技术产业贡献持续提升，全市高新技术产业总产值达949.61亿元、增长了1.44倍，占比提升至22%。先导性产业发展态势持续提升，石墨烯、增材制造、膜材料等一批先导性产业项目率先布局，产业发展加快向中高端迈进。企业发展质态持续提升，全市规上工业企业达2549户、累计增加527户，产值过亿元企业翻了一番、达910户，新增新三板上市企业17户。“三降”，即高耗能行业产值增速下降6.8个百分点，高耗能行业投资占比下降5.8个百分点，单位工业增加值能耗下降27.7%，经济生态化建设取得积极成效。服务业发展提速快进。服务业增加值增速连续四年高于GDP增速。旅游业蓬勃发展，项王故里、三台山森林公园等一批龙头型景区建成运营，三台山森林公园创造了当年开工建设、当年开园运营、当年获批国家4A级景区的奇迹，衲田花海获评江苏“四季赏花地”，日最多接待游客超过7万人；创成国家4A级景区6个、总数达9个；2016年接待游客近2000万人次，旅游收入突破200亿元，增速双双超过20%，“景点旅游”向“全域旅游”的转变逐步加快。电子商务异军突起，成功举办中国“互联网+创业”大会、第四届中国淘宝村高峰论坛，获批江苏省跨境电子商务试点城市、创建全国网络市场监管与服务示范区。8个电子商务产业园累计入驻企业550户，其中宿迁电商产业园引进京东、途牛、当当等11家全国50强互联网电商企业、累计入驻企业达252户，获批国家电子商务示范基地，荣膺“优秀电子商务园区20强”。投资超百亿元的京东信息科技园、京东云华东数据中心等项目建成投运，全市电子商务交易额年均增长85%以上，电子商务产业发展创造了备受瞩目的“宿迁现象”。金融服务体系更加健全，南京银行、民生银行等4家银行成功引进，南京证券、海通证券等9家证券机构先后入驻，太平财险、华泰财险等11家保险公司开业运营，各类金融机构超过120家、翻了一番；大地、人保等保险公司电商中心和呼叫中心相继落户，保险小镇初具规模；存贷款余额分别达2207.43亿元、1960.37亿元，分别增长79.9%、95.5%，金融业对经济发展的保障作用不断增强。农业现代化水平稳步提升。粮食生产实现“十三连丰”，四大特色产业产值占比达82%，设施农业、设施渔业占比分别提升至17.2%、26.7%，农业机械化水平达81%，农业生产方式加速向集约化转变。省级以上农业龙头企业达48家、其中国家级5家。积极发展“一社一群一中心”，建成家庭农场集群及综合服务中心41个，家庭农场总数达6561家、居全省首位；深入实施“互联网+”农产品营销行动计划和“一村一品一店”提升行动，农产品网店突破3万家，“一村一店”实现行政村全覆盖，农业转型发展进一步加快。创新驱动逐步增强。实施“五大行动计划”，累计引进国家“千人计划”专家92人，入选省“双创人才”69人，人才总量达45万人，宿迁对各类人才的吸引力持续提升；建成苏北工业技术研究院等10家产业技术研究院，规上高新技术企业研发机构建有率达100%，全社会研发经费支出增长1.32倍、占GDP比重提升至1.54%，科技进步贡献率超过50%，创新驱动正加快成长为引领宿迁转型发展的新引擎。

五年来，我们一点一滴强功能、塑特色，城乡面貌焕然一新。按照“生态、精致、时尚，充满人文关怀”的城市建设定位，坚持从点滴小事做起，从功能特色着力，全面推进新型城镇化。全市城镇化率达57.53%、提高6.56个百分点，中心城市建成区面积扩大到85平方公里、人口增至75万。城市个性特色愈发鲜明。围绕林荫、彩色、花园、海绵“四个城市”建设目标，着力推进景观、生态、休憩相融合的复合型绿化。林荫城市建设上，先后建成世纪大道、奥体路等70余条林荫路，市区主次干道绿化普及率达100%，林荫路推广率达71%。彩色城市建设上，实施了西湖东路、九鼎公园等80余项城市彩化工程，打造了洞庭湖路、千岛湖路等一批彩色景观道和落叶景观路，春花烂漫、夏荫浓郁、秋色绚丽、冬景素雅的城市形象全面展现。花园城市建设上，规划建设了国土西巷爱情隧道、骆马湖路南侧沿墙绿带等近30处花海花墙花廊，雄壮河湾、印象黄河等一批公园陆续开放；按照民生园林的理念，率先推进11个单位庭院开放式改造，建成了人民大道两侧、项王东路两侧等40余处街头游园，形成了花香迎宾、绿树绕城、庭院缤纷的城市特色。海绵城市建设上，先后建成西湖路运河桥桥头公园、财政广场、鸣凤漙公园等一批海绵城市试点项目，普通绿地变身为有生命、会呼吸的城市海绵体。中心城市累计新（改）建绿地5.6万亩，绿地率、绿化覆盖率分别达39.7%、42.7%，分别居全省第七、第八。央视《新闻联播》对我市民生园林进行了专题报道。城市营建机制不断优化。创新城市建设模式，探索“策创营”前置，推行EPC、PPP等模式，城市建设运营水平不断提升。创新特色塑造模式，建立园林绿化项目具美评审机制，建成下相公园、黄河南路花街等一批精品工程，城市更具美感、更加精致。探索商业发展模式，积极培育独具特色的业态和街区经济，规划了西楚文体街区、新盛街历史文化街区，建设了仁恒里等一批特色鲜明的街区，城市活力得到进一步提升。城市功能配套日趋完善。从打基础、利长远的工程干起，新建和改造环城西路、黄河南路、青海湖路等一批城市主次干道，市区“十一纵十二横”路网结构基本形成，路网密度提升了16.4个百分点；实施“雨污分流”，打通城市排水“经络”，累计铺设雨污水管网1470公里，实现雨污分流面积130平方公里。大力推进高清监控“四大工程”，织密防护“天网”。促进城市商圈繁荣，苏宁广场、水韵城等一批城市综合体开业运营，群众多层次购物需求得到更好满足。从矛盾多、问题大的地方抓起，改造老旧小区32个，完成市区213个小区物业整治提升，市民居住环境进一步优化；实施主街道城市形象提升、道路通达、摊点集中疏导区建设“三项工程”，规范提升店招店牌和城市家具，8条精品路街景焕然一新，整改城市断头路、盲道缺失等道路不畅问题636处，3个大型摊点集中疏导区建成投用。从惠民生、增民利的小事做起，深入实施中心城市民生“六项工程”，扎实推进“两场一街一中心”建设，新（改）建停车场24个、标准化菜市场17个、特色餐饮街区6个、社区服务中心和综合服务体125个，打造了15分钟便民生活圈；建成遮阳棚、跳蚤市场等一批便民设施，新增公共停车泊位3.58万个，市区公厕全部达到三类以上水冲式公厕标准且24小时免费开放，11座公厕入选2016年全国“最美公厕”。宿迁既拥有了中小城市的舒适度，也具备了大城市的功能性，日益成为一座宜居宜业的城市。城市文明程度持续提升。坚持大处着眼、小处着手，形成“订立规矩、重塑习惯、涵养文明”城市管理三部曲。严管重罚、树立规矩，以整治闯红灯为突破口，持续加大交通秩序、城市养犬等整顿力度，立起城市管理“宿迁规矩”。建章立制、养成习惯，制定实施店外经营、广场舞规范等一系列规章制度，推动“宿迁习惯”养成。文明涵养、培育自觉，出台《宿迁文明20条》，通过“小习惯”唤起市民的文明意识；创新开展“礼让斑马线·文明我点赞”活动，促进“宿迁习惯”内化为“宿迁文明”。深入实施市县联动，将市区成熟经验做法向县城延伸，县城建设管理水平得到大幅提升。沭阳“中国花木之都”、泗阳运河之畔最美县城、水韵泗洪特色品牌进一步打响。在苏北率先实现省级文明城市全覆盖。美丽乡村建设成效明显。新（改）建农村公路3805公里、桥梁425座，实施草危房改造1.79万户，新增农村无害化卫生户厕28.94万座，整治农村河道272条，农村环境不断优化。创成中国最美乡村3个、省三星级康居乡村50个、省“美丽乡村”23个，建成一批乡里中心和公租农庄，镇村风貌进一步提升。重大基础设施加快推进。宿淮铁路、宿新高速、205国道、245省道等建成通车，徐宿淮盐高铁开工建设，合宿新高铁列入国家路网规划，宿迁即将迈进高铁时代；运河宿迁港主体工程完工，古泊河船闸、成子河船闸等建成通航，公铁水一体化的交通运输体系初具雏形，宿迁人民的“赶海梦”更近了。建成全省第三、苏北首个全光网城市，国电二期项目加快推进，以500千伏双泗变、龙湖变、沭阳变、项王变为支撑，220千伏中心城区和三县双环网为骨干的供电网架基本形成。重大基础设施的不断完善，极大地方便了群众生产生活，也为长远发展提供了坚实支撑。

五年来，我们标本兼治抓源头、优生态，绿色发展成效日益显彰。坚持源头管控与制度约束并进、污染防治与生态建设并重，生态文明建设水平加快提升，环境质量持续向好。生态基础设施不断完善。新（扩）建城市污水处理厂6座，日污水处理能力达51.75万吨、提高了0.85倍；垃圾焚烧发电厂二期、有机生物处置中心、危险废物填埋场等先后建成，生活垃圾转运处置体系基本建成。生态环境整治扎实开展。推进大气治理，全面落实“气十条”行动计划，积极开展扬尘、工业废气、燃煤锅炉等专项整治行动，秸秆禁烧和综合利用水平稳居全省前列。市区环境空气质量优良率比2013年提升10.2个百分点，PM2.5年平均浓度下降25.1%，三县空气质量优良率稳步提升。推进异味治理，按照“限期治理一批、停产整治一批、取缔关闭一批”的要求，全面开展“一企一管”“一企一测”“一企一治”，对化工企业进行逐个整改，有效遏制了化工园区有机废气和无组织气体污染。推进水体治理，按照全域规划、全域设计、全域配套、全域修复、全域清流“五全”治理思路，对西民便河等16条城市河道进行全面整治，打造了水清林绿的生态走廊；国考断面水质达标率高于全省平均水平。推进土壤治理，启动土壤污染防治方案编制工作，完成耿车废旧物资加工区域土壤污染现状初步调查，为治理土壤污染提供了科学依据；加大农业面源污染整治力度，农业废弃物综合利用率提高到90%。植树造林工程持续实施。新造成片林41.89万亩，林木覆盖率提高到30.1%、位居全省第二；开展“杨树更新改造年”活动，着手解决杨树飘絮问题；创成国家级生态乡镇16个、省级46个，绿色生态成为宿迁最靓丽的城市名片。生态环保机制更加健全。编制生态红线区域保护规划、骆马湖和洪泽湖生态环境保护总体规划。出台“1+1+N”文件，率先建立行政执法与司法联动的生态保护机制。全面推行“河长制”。率先开展排污权有偿使用和交易试点，实施污染企业关停并转，全市单位GDP能耗、水耗分别较2012年末下降了4.1%、38.5%，化学需氧量、二氧化硫等指标超额完成省下达的“十二五”减排任务。

五年来，我们不等不靠推改革、求突破，发展活力持续增强。以行政审批制度改革为主要领域，以“减权去利便利化”为主要要求，持续推动经济体制、农业农村、社会事业等领域改革，市场和社会主体活力加快释放。经济体制改革持续深化。围绕降低准入门槛，全面放宽登记注册条件，前置审批项目由199项压减至37项，实现市级基本无前置审批；完成53项市级工商登记后置审批事项清理，“办照容易办证难”得到有效破解，全市新登记私营企业5.8万户、注册资本1935.39亿元，较改革前分别增长46.15%、138.47%。围绕优化办事流程，创新推出“并联审批”“并联+串联”“多图联审”“多评合一”等审批模式，开展“四证（照）一体”“三证合一”“一证一号”等改革，探索“互联网+”电子商务审批服务新机制，政务服务综合效能提高70%。围绕企业降本增效，在全国率先开展资格资质去行政化改革，569项资格资质项目仅保留53项，有效激发了社会主体活力；在全省率先实施资源要素价格改革，对水电气热大用户实施合同交易和水电气热用户工程市场化改革，让企业有了“讨价还价”的权利；探索推进工业类产品生产许可制度改革，在全国率先对部分工业类产品暂停行使生产许可和实施先证后核制度；在全省率先启动垄断性服务价格改革，市区取消了自来水过户费、用电移表费等15项不合理收费，免收了有线电视初装费、土地使用权交易服务费等8项收费，放开普通公路、城市道路车辆救援服务收费等14项政府定价管理，企业生产成本大幅降低、创新活力有效激发。围绕社会协同治理，全面推行“五放五减十鼓励”改革办法，推动社会组织自主办会、独立运作，全市登记社会组织5741个，较改革前增长超过一倍；完善社会信用体系基本构架，累计归集数据超过1.6亿条，跃入全省信用建设第一方阵，成为全国首批创建国家社会信用体系建设示范城市。围绕事中事后监管，建立“五位一体”综合监管体系，在全国率先搭建集“并联审批”“联动监管”于一体的证照联动监管平台，推动监管信息互联互通；全面推行“双随机”监管，变过去人盯人监管模式为信息化系统监管。农业农村改革持续深化。深入推进农村土地承包经营权确权登记颁证工作，以县为单位在全省率先基本完成确权登记颁证；率先开展村级土地股份合作社、农村承包土地经营权抵押贷款和涉农贷款保证保险试点，率先实现县级农村产权交易平台全覆盖，累计完成交易3736笔、交易金额25.27亿元，均领先全省。社会事业改革持续深化。医疗卫生改革深入推进，市第一人民医院全面运营，二级以上医院增至32所，其中三级医院增至5所；同步推进乡村卫生机构一体化管理和新农合结报体系延伸到村工作，实现基本药物制度城乡全覆盖，群众基本就医负担明显减轻，具有宿迁特色的医疗服务体系初步建立。市公共卫生服务中心主体工程竣工，基本公共卫生服务项目人均经费标准提高到50元/年；实施管办分开、购买服务的乡镇卫生事业改革，开展村居卫生机构达标建设，政府购买基本公共卫生服务乡镇超过三分之二，全市1310个村卫生室和112个社区卫生服务站达到标准化要求，形成了公共卫生服务政府织网兜底的责任体系。教育改革稳步推进，实施中心城区学校管理体制调整工程，形成了中心城区普通高中由市统一举办管理、义务教育和学前教育由区举办管理的格局。出台深化民办教育改革与发展意见等政策措施，民办教育发展环境进一步优化。制定提升学前教育公共服务水平意见，确立了政府提供非营利性、基本性学前教育公共服务，社会力量提供具有选择性的学前教育服务的职责定位。积极推进绿色校园建设，努力营造功能齐全、绿色环保、舒适安全的育人环境。

五年来，我们放眼四海广交流、促合作，对外开放成果丰硕。牢固树立“四海”意识，持续扩大对外开放，城市开放度和竞争力进一步增强。招商引资成绩显著。创新“三位一体”招商机制，紧盯长三角、珠三角、港台等重点区域，举办宿迁经贸洽谈会、“接轨上海”系列活动等重大招商活动40余场，格力电器、海天调味品、福能科技、海欣申禾纺织等一批计划投资超10亿元的大项目成功落户，可成科技连续5年增资、共投资18亿美元，累计引进亿元以上开工项目725个、竣工607个。以德国为重点的对欧招商局面全面打开，连续三年赴德举办经贸合作论坛，美宿德创材料、科曼公司等一批知名企业相继落户。累计使用外资23.99亿美元、新批外资项目296个。外经外贸稳步增长。与165个国家和地区开展经贸往来，外贸备案登记企业达2338户，累计进出口151.7亿美元；积极推动宿迁本土企业“走出去”，累计完成境外投资3.6亿美元，超过前15年总和，经济外向度进一步提升。载体建设提档升级。围绕“五有三转”要求，不断提升各开发区承载能力，全市开发区累计竣工亿元以上项目564个。宿迁及沭阳开发区创成国家级经济技术开发区，宿豫开发区创成省级高新技术产业开发区，6家共建园区综合考评持续位居全省第一方阵、苏宿园区连续七年名列第一，规划建设8个先进特色产业园区，为外向型经济跨越发展提供了有力支撑。国际合作迈上新台阶。与新西兰因弗卡吉尔市、德国诺伊维德市缔结友好城市，与意大利摩德纳市、加拿大坎贝尔河市签署友好合作备忘录，与印度、哈萨克斯坦等10多个国家的城市建立良好关系，国际友城数量从3对拓展到5对，诺伊维德市荣膺“对华友好城市交流合作奖”、成为首个获此殊荣的宿迁友城，宿迁的国际影响力不断增强，“国际风范”愈发彰显。

五年来，我们矢志不渝惠民生、促和谐，百姓福祉稳步提升。紧紧围绕问题和需求导向，着眼公平普惠、成果共享，全力做好群众最关心、最急需的民生实事，群众获得感更实更足。省最新“六大体系”监测报告显示，我市就业服务、基本医疗卫生、终身教育3个体系发展指数均居全省第一，民生幸福“六大体系”总发展指数全省第一。群众就业更加充分。进一步完善公共就业服务体系，累计城镇新增就业22.67万人、农村劳动力转移就业19.95万人，城镇登记失业率实现“五连降”。坚持以创业带动就业，累计建成市级以上各类创业载体27家，扶持8.44万人成功创业，带动就业33.2万人。连续举办三届“金种子”网络创业大赛，“中国淘宝镇”“中国淘宝村”数量分别占全省三分之一、四分之一，平均每13人就有1人从事互联网创业及关联产业，“轻点鼠标”成为群众增收致富的新路子。社会保障更加有力。城镇职工“五险”扩面新增累计达136.26万人次，城乡低保标准提高到490元/月和390元/月。大病保险实现全覆盖，城乡医保住院补偿比例稳定在75%以上，社会救助体系基本建成。城市社区居家养老服务中心基本实现全覆盖，农村社区居家养老服务中心覆盖率达87%，每千名老人拥有床位数达44.2张、位居苏北第一，在全省率先推行失能五保老人集中供养制度，养老产业发展进一步加快。社会事业更加繁荣。三县两区全部通过全国义务教育发展基本均衡县（区）创建认定；高考本科录取率达55.51%，较2012年提高22.9个百分点；宿迁学院成功申本，中心城区“八校”建设扎实推进，D级校舍全面拆除并重建到位，越来越多的孩子享受到更加优质的教育资源。“暖心”工程持续实施，购置20余万条床垫、3.5万台套饭菜保温设施，让孩子们住得更温暖、吃得更热乎。完成棚户区改造6.06万户，建成各类保障性住房13.4万套，货币化安置率全省第一。市区公交车数量和公交线路分别增长1.9倍、1.1倍，新增空调公交车460辆，候车时间缩短至10分钟左右，乘坐公交车人次突破1.5亿；先后建设279个站点、投放6350辆公共自行车，累计借车361.14万人次；镇村公交开通率达100%，在苏中苏北率先实现镇村公交全覆盖，城乡居民出行更加便捷。群众生活更加多彩。市博物馆、图书馆、档案馆建成使用，宿迁音乐厅成功运营，文化建设工程综合指数跃居苏北首位；成功举办书画作品进省展、晋京展，6人次入选中国书法兰亭奖，30余件作品入选全国美展，6部文艺作品获省第九届“五个一”工程奖。市青年乐团、拂晓艺术团、大拇指艺术团揭牌成立，促进了文艺事业的活跃。龙王庙行宫和中运河宿迁段被列入《世界遗产名录》，新增国家级文物重点保护单位3处、国家级非遗名录项目1个，越来越多的“宿迁记忆”得到保护和传承。全民健身运动广泛开展，“10分钟健身圈”提档升级全面完成，市体育中心和全民健身中心投入使用，宿迁籍运动员在里约残奥会斩获5金3银、金牌数居全省第一，国际生态四项赛等体育赛事精彩纷呈。扶贫开发更加深入。国家扶贫改革试验区建设全面推进，“三大片区”综合开发扎实开展，“挂村包户”工程深入实施，逐步减免低收入农户医疗、子女上学及其他需要承担的费用，脱贫基础进一步夯实，99.5万低收入人口人均年收入如期达到4000元标准，360个省定经济薄弱村基本实现新“八有”，全面完成“十二五”脱贫攻坚任务。平安创建、综合治理、信访稳定工作成效明显，“六五”普法获得省政府表彰，安全生产工作连续十一年荣获省目标考核优秀奖，公众安全感排名全省前列。民防、物价、气象、地震、食药品、宗教、侨务、人民武装和双拥、档案、党史、地方志等工作实现了新进展，妇女、儿童、青少年、老龄、科协、红十字、慈善等事业取得了新进步。

五年来，我们旗帜鲜明讲政治、守规矩，自身建设不断加强。始终把遵守政治纪律和政治规矩摆在第一位，扎实开展党的群众路线教育实践活动、“三严三实”专题教育和“两学一做”学习教育，政府执行力和公信力显著增强。依法行政深入推进。强化行政复议规范化建设，建立健全新型政府法律顾问制度和市长约谈、行政执法、行政败诉追责“三项机制”，让施政方式、治理方式更加规范、公开。自觉接受市人大及其常委会法律监督和工作监督、市政协及社会各界的民主监督。廉政建设全面加强。严格落实中央“八项规定”、国务院“约法三章”、省委“十项规定”和市委“实施意见”，深入开展专项整治，严厉查处和纠正不正之风，全面完成领导小组撤销、首轮公车改革等各项任务，“三公”经费持续压降。严格落实“一岗双责”，不断强化政风行风建设和审计监督，形成了良好的政风新常态。服务效能大幅提升。完善公开承诺、首问负责等制度，实行重大项目挂钩帮扶机制，深入开展“三服务三促进”“两访两帮”，营商环境进一步优化。深化“群众不满意”事项整治，加大行政问责力度，严厉查处不作为、慢作为，政务环境不断优化。深化政务服务云审批，加快政务云服务“一张网”建设，实现信息多跑路、群众少跑腿。2016年市便民方舟完成行政审批办件5.76万件、同比下降47.9%，公共服务办件239.93万件、同比增长12.5%，政府职能转变进一步加快。“网上宿迁”荣获中国政府网站绩效评估“优秀”等级，“宿迁之声”微博微信位列人民日报政务指数排行百强，“速新闻”“手机台”成为全国具有较强影响力的地方新媒体。

各位代表，回首过去五年，世界经济持续低迷，宏观形势急剧变化，问题矛盾急剧增多，经济下行压力不断加大，经济社会发展面临的挑战和压力前所未有，遇到的困难和考验前所未有。在这样的形势下，宿迁经济和社会事业仍然保持了稳步向前、稳中有进的健康发展势头，取得了辉煌的业绩，实属不易、难能可贵。这主要源于我们坚持以“自我改革”精神创新体制机制，以“巡更精神”提升服务水平，营造了宿迁良好的“小气候”，其中的宝贵经验值得总结：一是坚持科学发展，质量速度相统一。牢牢把握宿迁做大总量和转型升级双重任务，既立足于“快增”，推动经济增长在合理区间内能快则快，始终保持一个能够支撑事业发展需要、满足居民持续增收，真实的、不含水分的合理速度；更专注于“快转”，积极转变组织领导经济工作方式，促进产业发展向中高端、集聚化迈进，致力从跟随模仿向先导引领转变。二是坚持尊重市场，锐意改革激活力。始终遵循市场规律、经济运行规律和社会发展规律，准确把握政府与市场关系，坚持用市场化的理念和方式推进经济工作，用改革的思路和办法破解瓶颈制约，最大限度地减少对市场和社会主体的束缚，充分调动各方面的积极性，以政府权力的减法换取市场活力的加法。三是坚持遵法以动，善治善为用好权。将行政行为纳入法治框架内考量，规范重大行政决策程序，建立健全重大决策合法性、风险性、廉洁性审查评估制度，以及“三重一大”事项集体审议决策制度，决策机制进一步完善。建立重大项目民意征集、专家咨询论证、第三方调研评估等机制，开展技术路演，动态调整完善，提升行政决策的科学化水平。四是坚持担当有为，苦干实干强作风。始终把使命挑在肩上，把责任抓在手中，保持干事创业的激情、攻坚克难的勇气，敢于涉险滩，敢啃硬骨头，在担当中发展、在发展中担当。坚持干字当头，一个项目一个项目抓落实，一个环节一个环节抓推进，做到善始善终、善做善成。五是坚持倾听民意，政民互动聚合力。始终把民意作为最大的执政资源，将主动公开当成一种责任、一种义务，围绕群众关心关注的重大决策、重要政策，通过“您知道吗”栏目、“鼎鼎有民”网站等进行深度解读，让公众更好地了解和掌握。把沟通互动作为一种习惯，常态化举办“星期三政民互动”等活动，从公众的不满意和期盼中探寻决策重点和努力方向，形成了全社会凝心聚力、共谋发展的良好氛围。

各位代表，过去的五年，我们共同谱写了宿迁发展史上的新篇章。这些良好业绩的取得，离不开省委、省政府和市委的正确领导，离不开省级机关和苏州等兄弟市的关心帮助，离不开市人大、市政协和社会各界的监督支持，离不开全市人民的团结一心、奋力拼搏。在此，我代表市人民政府，向奋战在各行各业的全体劳动者，向人大代表、政协委员、各民主党派、工商联和无党派人士、各人民团体，向离退休老同志，向中央和省驻宿单位以及人民解放军、武警官兵、公安干警，向参与宿迁建设的海内外客商、广大援宿干部，以及所有关心支持宿迁发展的各级领导、各界人士，致以崇高的敬意和衷心的感谢！

在肯定成绩的同时，我们也清醒地看到，全市经济社会发展中还存在一些问题和短板：经济下行压力较大，部分指标增速回落；产业层次不高，实体经济困难较多，转型升级依然任重道远；基础设施还有短板，人气商气不够集聚，中心城市辐射带动能力亟待提升；资源要素制约加大，节能减排压力增加，生态保护与经济发展需要更好统筹；公共服务体系不够完善，城乡居民持续增收难度较大；政府职能转变有待加快，新常态下组织领导经济工作方式尚需改进，等等。对此，我们一定高度重视，采取更加有力措施，认真加以解决。

未来五年政府的工作思路和主要目标

未来五年，我们肩负着全面建成小康社会的重大历史使命。纵观国内外形势，全球经济还处在深度调整中，宏观环境仍趋复杂多变，经济下行压力依然较大，经济发展面临的复杂性、不稳定性有可能进一步增加，推动我市各项发展还有很多不确定性。然而，世界新一轮科技革命和产业变革正加速推进，我国经济发展长期向好的基本面没有变，“一带一路”、长江经济带、江苏沿海开发等战略的深入实施，为我们带来了多重叠加的发展机遇；党中央、国务院的坚强领导，给了我们克服困难的坚定信心和决心；省委、省政府加大对苏北发展的支持，将为宿迁发展注入强劲动力；市第五次党代会描绘了宿迁未来发展的美好蓝图，极大地激发了全市人民的干劲和热情。我们将主动适应把握引领经济发展新常态，找准路径、精准发力，全力推动经济社会持续健康发展。

今后五年政府工作的指导思想和目标是：高举中国特色社会主义伟大旗帜，全面贯彻党的十八大和十八届三中四中五中六中全会精神，以马克思列宁主义、毛泽东思想、邓小平理论、“三个代表”重要思想、科学发展观为指导，深入贯彻习近平总书记系列重要讲话精神，认真落实省第十三次党代会部署，按照市第五次党代会要求，统筹推进“五位一体”总体布局，协调推进“四个全面”战略布局，精准发力落实“五大发展理念”，精准发力落实“两聚一高”，精准发力推动供给侧结构性改革，精准发力实施生态立市、创新驱动、产业强市、城乡协调、民生优先五大发展战略，精准发力提高全面建成小康社会的质量和水平，突出生态富民，加快转型发展，努力建设经济强、百姓富、环境美、社会文明程度高的新宿迁，谱写好中华民族伟大复兴中国梦的宿迁篇章。

——建成发展更有质量的新宿迁。到2021年，地区生产总值比2012年翻一番，达到3300亿元，人均地区生产总值达到67000元；二三产业比重达到90%，四大特色产业、四大新兴产业规上企业主营业务收入分别突破4000亿元、700亿元，现代农业发展水平指数达到90%。

——建成百姓更得实惠的新宿迁。民生实事项目深入实施，社会保障体系不断完善，精准扶贫精准脱贫任务全面落实，实现居民收入增长与经济增长同步、劳动报酬提高与劳动生产率提高同步。到2021年，城乡居民人均可支配收入比2012年翻一番，达到27600元，广大人民群众生活水平进一步提高。

——建成城乡更加协调的新宿迁。新型城镇化和城乡发展一体化扎实推进，市域城镇体系更趋健全，中心城市首位度明显提高、辐射带动力日益增强，常住人口城镇化率达到62%。三个县城的能级不断提升、功能更加完善，特色城镇和美丽乡村建设深入开展，城乡发展差距进一步缩小。

——建成生态更具优势的新宿迁。国家生态文明建设示范市创建扎实开展，生态经济示范区建设深入推进，环境质量明显改善，绿色生态产业体系初步形成，生态宿迁的城市品牌进一步打响。到2021年，全市城镇绿化覆盖率达到40%，林木覆盖率达到31%，空气质量二级标准的天数比例达80%以上。

——建成社会更为和谐的新宿迁。社会主义核心价值观深入人心，文明创建成效全面提升，创成全国文明城市、全国社会信用体系建设示范城市，宿迁“文明20条”成为行动自觉，文明有礼、崇德向善的社会风尚更加浓厚。公共文化服务体系日益完善，公共安全体系更加健全，社会公平正义得到有效保障。

实现上述目标，需要我们牢牢把握稳中求进这一工作总基调。稳中求进工作总基调既是治国理政的重要原则，也是做好经济工作的方法论。“稳”是主基调、是大局，重点是稳定经济运行、稳住市场预期、稳住社会大局，坚决守住社会民生、生态环境等底线。“进”是目的、是指向，重点是突出转型升级的主攻方向，加大结构调整力度，在产业集聚升级、培育新的经济增长点、深化科技创新驱动等方面取得较大进展，在推动深化改革、民生改善、社会治理等关键领域有新的进步。总的来说，就是在稳的前提下奋发有为、不断进取，充分做实居民收入、企业利润、财政收入“三个口袋”，全面推进人口集聚、要素集聚、产业集聚“三个集聚”，坚定不移发展工业经济、城市经济、农业经济“三大经济”，努力推动全市经济社会持续健康稳定发展。

全力做好2017年政府重点工作

2017年是新一届政府任期的第一年，也是全面建成小康社会的关键之年。我们将全面落实党的十八大、十八届三中四中五中六中全会和习近平总书记系列重要讲话精神，深入贯彻中央经济工作会议、省第十三次党代会和经济工作会议部署，按照市第五次党代会要求，统筹推进“五位一体”总体布局，协调推进“四个全面”战略布局，坚持稳中求进工作总基调，牢固树立和贯彻落实“五大发展理念”，主动适应把握引领经济发展新常态，深化供给侧结构性改革，以“三抓三促”为主线，以“三转三稳”为取向，以提高发展质量和效益为中心，聚力创新、聚焦富民，全面做好稳增长、促改革、调结构、惠民生、优生态、防风险各项工作，促进经济平稳健康发展和社会和谐稳定，在高水平全面建成小康社会上迈出坚实步伐。全市经济社会发展的主要预期目标是：地区生产总值增长8-8.5%，一般公共预算收入同口径与去年基本持平，固定资产投资增长13%左右，社会消费品零售总额增长12%，全体居民人均可支配收入增长与经济增长同步，节能减排完成省下达的目标任务。重点抓好六个方面工作：

（一）以集聚升级推动产业转型，创造更有前景就业岗位富民。

加快制造业集聚，做大总量促转型。当前宿迁最大问题仍是发展不充分，加快发展、做大总量依然是首要任务，做大做强工业制造业是完成这一任务的最有效路径。我们将紧紧围绕市域“4+4”、县区“2+1”产业定位，全力以赴抓集聚、上规模，抓总量、促转型。聚力招商引资，扩大项目增量。立足“三位一体”招商，加强专业招商队伍建设，用好“宿商”资源，强化以商引商、中介招商、委托招商等。建立“一站一库三网”对德招商网络，巩固对台招商优势，紧盯长三角、珠三角等重点地区，精心组织好2017中国宿迁经贸洽谈会、中国宿迁（德国）经贸合作论坛和“接轨上海”系列活动。围绕主导产业，重点招引延链、补链、强链项目，加速产业集聚，年内力争引进主导产业项目占比超过70%。壮大新兴产业，培育新增长点。立足“3+3”产业基地，加快建设激光产业园、膜材料等8个先进特色产业园区，大力发展高端引领型项目，积极培育石墨烯、增材制造等先导性产业，推动智能家电、绿色建材等新兴产业快速壮大。加快项目建设，集聚发展动能。围绕扩大有效投入，积极提升项目建设层次，优化投资结构，从源头上提高发展质量。扎实落实“五个一”推进机制，确保鑫博高分子材料、华工激光等170个新型工业化项目按序时进度推进。发展外经外贸，扩大对外开放。引导更多的企业“走出去”，支持企业参加广交会、华交会等各类展会，年内培育进出口超亿美元企业2户、超千万美元企业50户。做强园区平台，提升载体能级。按照“五有三转”要求，支持泗阳开发区争创国家级经济技术开发区，宿迁高新区争创国家级高新技术开发区，宿迁经开区、宿城开发区争创省级生态园区。加快综保区建设，年内力争取得实质性突破。

推动质态提升，优化存量促转型。加快改造提升优势传统产业，积极推进技术创新，认真落实“五大任务”，进一步提高产业发展质态、增强综合竞争力。提升传统产业。传统产业不等于落后产业，其在吸纳就业、提供税收等方面具有不可替代的优势，必须沉下心来做实做强。围绕食品饮料、纺织服装、机电装备、家居制造等传统产业，有效集聚原料、公共技术服务平台、整机等关键项目，逐步建立从原料生产到终端消费在内的全产业链，切实提升产业协作水平；高度重视传统产业转型升级，持续推进装备更新和技术进步，促进产品更新换代，不断增强产业市场竞争力。深入实施“511”工程。抓好核心技术产业化、信息技术应用等环节，支持企业兼并重组、技术改造，引导企业股改上市，推动企业规模发展，确保完成三年目标任务。强化职业经理人队伍建设，搭建企业人才引进、培训、交流新平台。突出创新驱动。加快各类科技创新平台建设，支持秀强、双星、斯迪克等创新型企业建立国家级研发机构，提升自主创新水平。借助宿迁技术交易服务中心发展科技中介服务产业，激励企业家探索创新要素重组，加速创新成果转化。年内创成苏北首家全国质量强市示范城市。落实“五大任务”。在坚决完成去产能、去杠杆、降成本、补短板任务的同时，着力在去库存上下功夫，按照“七优”要求，严控土地出让，出台促进中心城市房地产转型升级的具体办法，推动房地产业持续健康发展。

发展现代服务业，优化业态促转型。以重点项目和载体为主抓手，推动服务业提速发展，为产业转型积蓄更多动能。做精旅游产业。以PPP模式推进项王故里三期项目、三台山衲田花海配套提升工程，加快三台山森林公园微电影小镇、健康养生区建设。规划建设影视基地，推动影视全产业链发展。创建骆马湖—三台山国家级旅游度假区，三台山森林公园创成国家级森林公园，推动洪泽湖湿地景区创建国家5A级景区和国家级生态旅游示范区，支持沭阳花木生态旅游度假区创建省级旅游度假区，加快泗阳成子湖旅游度假区建设；湖滨新区加快发展旅游经济，并实现较大突破；洋河新区深入挖掘酒文化，进一步繁荣酒文化经济。做大现代金融。积极引进银行、保险、担保等传统金融机构，大力招引创投、风投、私募基金等新型金融机构，健全多层次现代金融体系。围绕建设保险创新综合试验区，探索“会省市”三方共建模式，加快保险小镇建设。争取组建地方金融资产管理公司等交易平台，成立市住房金融公司，进一步优化金融资源配置、丰富金融业态。

壮大网络经济，融合发展促转型。把“互联网+”贯穿到产业发展全领域，促进线上线下融合发展，为产业转型升级增添新动力。推动互联网+制造业深度融合。发展定制经济，加强与京东、阿里巴巴等知名电商合作，鼓励企业积极拓展定制生产业务。实施智能制造，依托京东（宿迁）物联网智能制造产业园，支持企业应用智能设备，加快装备智能化更新，推动制造业向中高端迈进。推动互联网+服务业平台建设。围绕“一区多点”功能定位，推动各类电子商务产业园扩模提质，加快京东、淘宝、苏宁易购等地方特色馆建设，抓好网络市场监管与服务示范区创建，发展共同配送、“体验式”消费等商业新模式，年内8个省级电子商务产业园实现交易额360亿元，增长50%以上。推动互联网+农业提档扩面。培育农村电商专业服务企业，完善农村物流通达体系，提升地方特色农产品网销市场占有率。继续推进“电商镇”“电商村”建设，年内“一村一品一店”建成率达65%。

（二）以活力提升推动城市繁荣，创造更宜居生活环境为民。按照“生态、精致、时尚，充满人文关怀”的建设定位，进一步完善功能配套，提升城市活力，努力打造让群众更有自豪感、幸福感、归属感的美好家园。

建设中央活动区，汇聚城市活力。中央活动区是融合中高端商务商业、文化、休闲、娱乐和居住等功能的城市核心区。以中央活动区建设为引领，加快提高城市空间环境品质，满足市民更高层次的需求，进一步提升中心城市首位度。提升两大商业圈层，促进中央活动区商业繁荣。加快以金鹰、宝龙、水韵城为主的第一商圈建设，推动从霸王举鼎广场、凤凰街区、新盛街到原河滨新村地块的第二商圈发展，形成人气、商气的集聚中心。支持宿豫区以“金三角”为龙头发展商业次中心、苏宿园区以苏式生活为风格更好地营建区域特色。做优街区业态，增强中央活动区特色魅力。加快建设充满青春活力的西楚文体街区、承载传统记忆的新盛街历史文化街区，完成仁恒里文创特色街区招商运营，规划散发市井烟火气息的凤凰街定制生活街区。推进城市家具改造，有序建设一批城市雕塑、文化小品，不断提升城市品位。策划系列品牌活动，增添中央活动区活力因子。挖掘本地风土人情、风俗习惯，打造具有宿迁“土味”的节日节庆。建设地方特色文娱活动载体，增强城市文化辨识度。进一步丰富衲田花海音乐会、衲田走秀、大剧院音乐季、项王故里开门大典等系列活动。加快时尚体育服务综合体建设，培育时尚体育“1+X+1”系列赛事，打造时尚体育城市。

精塑个性特色，彰显城市活力。以个性特色风貌打造独有的城市印记，使城市更具识别性。实施引湖入城，打造亲水之城。加快骆马湖宿迁大控制三角区退田还湖生态建设工程可研、报批等前期工作，推动中心城市由滨湖城市向亲水城市转变。深化“四个城市”，打造多彩之城。围绕林荫、彩色、花园、海绵“四个城市”建设目标，启动环城生态走廊建设，加快推进古黄河生态廊道、清风廊道等项目，建成环古黄河游步道，完成包括东关口公园在内的马陵河全域整治和景观建设；建设一批可进入的小游园、小绿地、小广场，继续推进中心城市单位庭院开放式改造，力争市民出行300-500米即可步入绿色空间。塑造特有风貌，打造魅力之城。明确城市总体色彩风格规划定位，制定建筑设计负面清单，加强建筑风貌管控，筹备“衲田杯”可持续设计国际竞赛，强化城市的地域特色、建筑风格等“基因”；加快如意黄花槐、千鸟园红枫林、山核桃林片区建设，着力塑造诗意乡情。设置招鸟留鸟饲喂设施，进一步丰富城市动物种类，促进城市生态系统平衡。

完善配套设施，支撑城市活力。围绕城市发展需要和群众生活需求，进一步增强功能配套。加快便民服务类配套设施建设。改造平安菜市、乾隆菜市等老旧菜市，完善停车场、第三代公厕、餐饮街等便民设施，抓好供电、供热、供气、供排水等市政设施建设，更好地满足群众生活需求。整合社区服务与公共设施职能，打造集停车场、菜市场、餐饮、便民服务等于一体的城市之家，年内永阳、南关、白堡等九个城市之家投入使用。加快功能提升类配套设施建设。实施交通改善工程，推进环城北路、三台山大道南延等22条道路和断头路改造，进一步增强道路通达能力；扎实抓好主街道形象提升，加大背街小巷环境整治力度，让城市更加整洁卫生、美观舒适；在城市绿地、街头空闲区域，合理配置休闲座椅、健身器材、遮阳棚等功能性设施，建设一批设施更齐全、服务更完善的城市公共空间。加快重大基础设施建设。推进合宿新高铁前期工作，加快徐宿淮盐铁路和综合客运枢纽建设，年内完成上海路快速化改造、宁宿徐宿迁收费站南移，推动宿连高速纳入省“十三五”交通发展规划，进一步织密区域交通网络。

优化城市治理，提升城市活力。推进城市治理标准化。探索建立城管非现场执法和信用管理机制，将农贸市场、流动摊点管理等纳入社会信用体系；完善城市规划建设管理导则体系，提高城市治理的标准化、法治化水平。推进城市治理精细化。精雕细琢街景容貌、建筑立面，持续开展店外店、占道经营等集中整治，改造提升老旧小区、老旧宿舍区，从细从小从实改善城市面貌，打造舒适宜居家园。推进城市治理联动化。继续推动市级建设管理理念和标准导则向县城延伸，实现市、县城市建设管理水平同步提高。加大《宿迁文明20条》宣传力度，巩固文明交通成果，提升城市文明水平，增强城市影响力。

（三）以供给侧结构性改革的理念推动“三农”发展，创造更有效发展方式惠民。围绕市场需求，以增加农民收入、保障有效供给为主要目标，以提升农业供给质量为主攻方向，以机制创新为根本途径，提高土地产出率、资源利用率、劳动生产率，促进农业农村发展向追求绿色生态可持续、更加注重满足“质”的需求转变。

推进“一挂三化”，为农村居民进城入镇开辟新路径。统筹谋划新型城镇化，强化“一挂三化”政策设计，利用增减挂钩节余指标省域内转让使用试点政策，与“两权”抵押贷款、农民住房贷款、“三权分置”等形成政策集成，撬动“三化”融合发展，促进农业向规模集中、工业向园区集中、居住向社区集中。实行差别化安置政策，充分尊重农民意愿，鼓励搬迁农民优先选择货币安置进城入镇。建立健全由政府、企业、个人共同参与的农业转移人口市民化成本分担机制，让进城农民享有均等的基本公共服务。

壮大现代农业，提升优质农产品供给水平。立足“四减四扩”目标，进一步健全农业产业体系、生产体系和经营体系。围绕种什么，加强产前规划和政策引导。不命令农民必须做什么，而是不轻易禁止农民做什么，做好规划引导和信息服务，促进农业结构调整。从市场需求和农民意愿出发，出台市场化引导政策，鼓励农民发展高效经济作物、特色水产、生态畜禽养殖，建设精准农业、设施农业、智慧农业。加大“三品一标”创建力度，不断提升农产品核心竞争力。围绕种得好，加强产中基础设施和服务配套。加大农田水利建设力度，推进区域规模化高效节水灌溉，协同推进高标准农田建设、中低产田改造、土地综合整治、农业综合开发、农村电网和道路桥梁改造等工程，不断提高农业抵御自然灾害能力和综合生产能力，为农业结构调整提供更好的配套服务。加强基层农技推广体系建设，促进农业科技成果转化；加大农业机械化推广力度，提升农业集约化发展水平；加快制定家庭农场集群综合服务中心运行导则，完善服务功能，提高服务水平。围绕卖得好，加强产后农业生产经营体系建设。建立健全农村冷链物流体系，积极培育冷链物流龙头企业；鼓励和扶持农产品精深加工，推动农业提质增效；引进培育农产品展示直销中心等实体交易平台，加强与京东、阿里巴巴等电商合作，大力发展农产品线上交易平台，加快推动农产品由“种得好”向“卖得好”转变。

建设美丽乡村，打造更好生产生活环境。优化镇村布局。依托自然条件和资源优势，挖掘农村民居特色元素，加快建设一批特色镇村。尊重农村居民居住习惯，严控农民集中居住区新建四层及以上住宅楼。完善功能配套。全面推动基础设施、公共服务向农村延伸覆盖，实施村庄环境巩固提升工程和水系沟通工程，塑造水清树绿、整洁有序的乡容村貌。扎实推进“杨树更新改造年”活动，提升乡土树种栽植比重。打造特色小镇。按照产业、建制、风情三大类别，因地制宜做好特色小镇规划，积极构筑产业发展载体和创新创业空间。

实施精准扶贫，让低收入群众口袋更快鼓起来。加快国家扶贫改革试验区建设，开展“挂村包户”等帮扶活动，年内实现15万低收入人口脱贫。实施贫困生扶贫助学工程，确保家庭经济困难学生不输在起点上。实施城市摊点疏导区贫困户孵化脱贫工程，帮助城市困难群体就业。针对因病、因残、因灾致贫等支出型贫困问题，在“扶贫+开发”、大病补充保险、残疾人照护体系等方面抓好政策落实，不让一村一户在全面建成小康社会进程中掉队。

（四）以减权去利便利化为核心推动重点改革，创造更灵活体制机制便民。围绕有利于增添经济发展动力、有利于促进社会公平正义、有利于增强人民群众获得感、有利于调动广大干部群众积极性，深入推进经济体制、农业农村、社会事业等领域改革，提高改革整体效能，扩大改革受益面。

坚持转变政府职能与激发市场活力并重，深化行政管理体制改革。加快行政审批制度改革。加大资格资质去行政化改革力度，减少职业资格审批要件，降低就业创业门槛。深化工业类产品生产许可制度改革，逐步压减先证后核类和暂时保留类产品目录。加快政务云服务“一张网”建设和移动终端APP开发，推进审批标准化、扁平化、便利化。加快企业投资项目“不再审批”、综合行政执法等改革，推广区域评审，提升审批效率。进一步压降制度性交易成本，年内实现2个工作日内办理发放市权范围营业执照，3个工作日完成市场准入，5个工作日完成不动产登记，施工许可和竣工验收均在50个工作日内办结。深入推进招投标改革。深化公共资源交易监管体制机制改革，加快开发全市统一的公共资源电子化交易平台，加强招投标全流程监管，切实解决围标、串标等问题。强化社会信用体系建设。加快全国信用示范城市创建，推进信用信息平台互联互通、信用信息归集，扩大信用产品应用范围，让信用的力量渗透到更多领域。深化资源要素配置改革。以土地、能源、融资、环境容量等为重点，加快推动要素资源配置的市场化改革，为企业创造更公平的竞争环境。

坚持提升改革“含金量”与增强群众“获得感”并举，深化社会事业领域改革。推进教育改革。支持社会资本在兴办高品质学校方面发挥独特优势，推动民办学校与知名企业联姻，进一步提升办学能力。更好履行政府的教育公共服务职能，努力实现教育普惠优质。启动义务教育阶段中小学教师“县管校聘”管理制度改革，加快教师由“学校人”变为“系统人”，推进教师资源县域内均衡配置，让更多学生享受优质公平教育。深化医疗卫生改革。支持社会办医提质，加快乡镇卫计改革，扶持乡镇医院健康发展。实施医疗机构间医学检验检查结果互认制度，建设医学专家会诊咨询服务中心，减轻群众看病就医负担。建立分级诊疗制度，引导基层医疗卫生机构与城市二、三级医疗机构建立双向转诊机制。开展医疗机构第三方评议，建立医护人员和医疗机构信用管理制度、医护人员宣誓制度，完善医疗机构监管办法，促进医疗机构规范、健康发展。

坚持保障农民土地权利与改进土地收益方式并行，深化农业农村领域改革。推进农村土地“三权分置”。开展土地经营权入股发展农业产业化经营试点，丰富土地经营权权能。深化农村集体产权制度改革。稳妥推进集体经营性建设用地入市，年内实现农村集体资产股份、农户宅基地使用权等交易品种零突破。探索建立农业农村发展用地保障机制，鼓励集体经济组织以出租、合作等方式，盘活农村存量集体建设用地、空闲农房及宅基地。推动农村“两权”抵押贷款增量扩面。完善确权颁证、配套服务、风险处置的闭环机制，探索抵押物价值评估和风险处置机制，更有效盘活农村资源。推动宅基地自愿有偿退出或转让，土地经营权依法自愿有偿流转，激活农民手中的“沉睡资产”。

（五）以新的路径推动生态文明建设，创造更美好绿色家园安民。围绕生态经济化、经济生态化，大力推进生态文明建设，加快提升绿色发展水平，实现产业竞争力和生态竞争力“双提高”，不断满足群众对环境和生活质量的新需求。

建设生态经济示范区。加强生态建设与环境保护，打通“绿水青山”向“金山银山”转化的制度通道。加快规划设计。挖掘宿迁生态禀赋和产业优势，同步推进空间规划和产业规划，培育新技术、新产业、新业态，提升创新能力和全要素生产率。完善基础设施。加强城镇污水处理设施和污水管网建设，加快城市污水处理厂提标改造。推进中德农村生活污水处置国际项目合作，探索农村污水处理新路径。改造垃圾收集容器、垃圾中转站等设施，年内新建道路（广场）果壳箱配置覆盖率达98%以上。推广城市餐厨垃圾PPP处理模式，年内市区餐厨垃圾处理场投入使用。加强废弃物处置设施建设，提升各种废弃物特别是危废的处置能力。加快建筑垃圾资源化利用平台建设，推动建筑垃圾消纳处置，年内资源化利用达2.5万吨。推进垃圾分类处理，年内确保全市垃圾分类覆盖面达到15%。调优能源结构。实施煤电机组节能减排与改造行动计划，提高天然气气化率，提升清洁能源在能源消费结构中的比重，年内实现10蒸吨及以下锅炉全部淘汰或实行清洁能源替代。推动集中热源点建设，提高集中供热覆盖率，降低燃煤散烧污染。推进试点先行。抓好8个生态经济示范镇建设，着力打造样板和示范，既加快生态建设，又促进经济发展，更提高人民群众生活质量。发展生态经济。加快淘汰“三高三落后”项目，大力发展生态工业、生态农业、生态旅游等绿色生态产业，实现经济效益、社会效益与生态效益的同步提升。

强化生态环境整治。全面落实“263”行动实施方案，重点推进三个方面的治理。推进水环境治理。开展两湖禁采、入湖河流整治、中运河沿线综合整治，启动沂南河、徐洪河、古山河、六塘河等河流整治，加快实施农村河道清流工程。推进大气环境治理。落实“气十条”行动计划，推进重点污染源整治，进一步提升空气环境质量。推进面源污染治理。强化畜禽粪污治理，推广综合利用技术模式，年内禁养区内养殖场（小区）完成关停搬迁。

完善生态保护机制。健全生态补偿机制。开展生态环境损害赔偿制度改革试点，完善河流生态环境损害经济调节机制，通过经济激励推动环境保护建设。推行市场化治理机制。发挥财政资金的引导作用，鼓励社会资金以PPP等形式参与生态文明建设。推广“DBO”模式，实施镇村污水处理项目试点，提升污染治理效果。加强环保信用监管。推动企业环保信用评价结果在行政许可、采购招标、信贷支持等工作中广泛应用，构建“一处失信、处处受限”的社会氛围。加大对新落地项目、已投资项目的契约管理，明确环境保护更为细致的民事责任，更加有力地惩处各类环境污染行为。

（六）以补强短板推动民生改善，创造更优质公共服务暖民。坚持把“老百姓的感受”作为衡量民生工作的标尺，进一步加大民生投入，认真办好“医教水住行”“法访文保收”等民生实事，让群众的“钱袋子”更鼓、获得感更多、满意度更高。

促进创业就业夯实民生之本。培育创业主体。深入开展“五小创业”活动，推进国家返乡创业试点地区建设。落实“创九条”扶持政策，完善市、县、乡三级全民创业服务体系。加大青年创业小额贷款发放力度，建设青年网络创业孵化基地。推进充分就业。实施家门口就业工程，完善就业援助制度，加强对农村转移就业劳动者的跟踪服务，促进就地就近就业。强化职业教育培训。充分利用现有职教资源，借助德国手工业协会、工业商业协会等培训资源，开展各类职业技能培训，培养更多高技能人才。

优化公共服务增进民生福祉。制定推行基本公共服务标准，对标找差、补齐短板。丰富教育发展内涵。加强寄宿制学校建设和管理，着力解决留守青少年、儿童的教育和成长问题。加快义务教育学校标准化建设，年内完成BC级校舍加固工程。提升卫计服务水平。加大公共卫生服务投入，年内基本公共卫生服务项目经费提高到60元/人/年。加快医疗卫生重点项目建设，市公卫中心、体检康复中心等项目年内建成运行。继续实施村卫生室提质工程，建立村医待遇增长机制。促进文化事业发展。加强“书香宿迁”建设，举办“文化四季”惠民系列活动，年内建成农村特色文化大院和文化广场50个以上。全年组织文艺演出、举办美术、书画展览等惠民文化活动不少于1000场。

完善社会保障做好民生兜底。增强保障能力。开展社保扩面“幸福行动”，实施“全民参保登记计划”。推进城乡居民医保统筹制度改革，实现医疗保险跨省异地就医联网结算。将孕期筛查纳入医保范畴，从源头上提高人口素质，避免贫困代际传递。建设幸福街区。加大棚户区改造力度，通过货币化安置、住房金融扶持等政策，年内改造2万户以上。完成市区10个老旧小区和自建宿舍综合整治、全市13座标准化菜市场建设任务。新（改）建城区公厕26座，完成农村改厕4.3万户。提升供水质量。加快推进区域供水“通村达户”工程，加强水源地生态保护，年内完成市区居民住宅小区二次供水设施改造。

强化社会治理夯实民生基础。加强社会治安。深化系列平安创建活动，实施社会治安防控体系“456”发展计划，推进公共安全视频监控系统建设，建立全市大数据社会治理联动服务中心，提升社会治理信息化水平。维护市场秩序。持续开展假冒伪劣、食品药品安全等专项整治行动，营造公平有序的市场环境。狠抓安全生产。深化企业落实安全生产主体责任专项行动，切实维护全市安全生产良好态势。抓好信访稳定。深入推进法治信访建设和“阳光信访”工程，努力营造和谐稳定的社会发展环境。

全面提升政府自身建设水平

新的机遇召唤我们励志前行，新的使命要求我们勇于担当。新一届政府将深入推进“两学一做”学习教育，不断加强自身建设，持续加快职能转变，努力开创政府各项工作新局面。

提高依法行政能力。把政府工作全面纳入法治轨道，加快建设职能科学、权责法定、执法严明、公开公正、廉洁高效、守法诚信的法治政府。坚持规范用权。高度警觉权力不当扩张、高度警觉权力借机牟利，积极推行政府法律顾问等制度，运用法治思维和法治方式推动工作，做到法定职责必须为、法无授权不可为。严格行政主体和程序。始终遵循市政府常务会、市长办公会等议事规则，按照重大行政决策程序办事，确保行政决策权限合法、实体合法、程序合法。全面落实行政执法责任制，坚持严格公正文明执法，规范执法行为，实现执法的法律效果与社会效果相统一。推动政务公开。充分发挥传统媒体、新兴媒体作用，进一步加大政务公开力度，让权力始终在阳光下运行。自觉接受人大及其常委会的法律监督、工作监督和政协的民主监督，重视社会公众监督和舆论监督，加强层级监督、行政监察和审计监督，确保各项工作在法律框架内开展。

提高服务发展能力。发挥政府作用的核心是更好而不是更多。新一届政府将大力发扬“店小二”精神，强化公仆意识和服务能力，进一步减少政府对微观经济的直接干预。从打造营商环境入手。按照有求必应、无事不扰、贴心服务的要求，深入开展“三服务三促进”活动，精心、精准帮助企业解决发展难题。制定出台营商环境评价体系和考核体系，进一步规范部门权力、优化制度供给，为实体经济创造低成本、便利、公平、安全的发展环境。保护企业产权和投资者合法权益，清除市场壁垒，促进各类要素自由流动和充分竞争。从提升自身能力入手。加强对新常态下经济运行新趋势、新特征、新变化的研究和学习，积极转变组织和领导经济工作方式，切实提高业务素质和水平，增强驾驭经济社会发展全局的能力。从开展“三进三帮”入手。强化宗旨意识、为民意识和服务意识，全面掌握农村状况，因村制宜、区别对待、精准施策，有针对性地制定工作方案和具体措施，帮助农民增收致富，促进农业农村更好发展。

提高执行落实能力。一分部署，九分落实。政府的各项工作，既需有系统、有组织的统筹谋划，更需抓落实、讲实效的有力执行。雷厉风行抓落实。强化立说立行的工作作风，以“等不起”的紧迫感、“慢不得”的危机感、“坐不住”的责任感抓好工作推进，确保各项工作任务快落地、早见效。勇于担当抓落实。大力发扬知难而进、迎难而上的担当精神，勇闯深水区，敢啃硬骨头，做到矛盾面前不回避、困难面前不退缩，在破除瓶颈、化解问题中推动发展。优化机制抓落实。健全督查考核机制，将决策部署与督办推进同安排、同部署，推动工作真落地、见实效。

提高廉洁从政能力。廉洁是政府公信力的基石。我们将按照干部清正、政府清廉、政治清明的要求，坚定不移地把政府系统廉政建设和反腐败工作不断引向深入。强化作风建设。从严落实中央“八项规定”、国务院“约法三章”、省委“十项规定”和市委“实施意见”，持续纠正“四风”。严格控制一般性支出，切实降低行政成本，把有限的资金更多地用在改善民生上。强化制度执行。认真落实党风廉政建设责任制和廉洁自律准则，推进反腐倡廉制度建设，建立健全廉政风险防控机制，把纪律建设贯穿于政府工作的全过程。强化执纪问责。深入推进政府系统预防和惩治腐败体系建设，严肃查处各类违法违纪案件，保持严惩腐败的高压态势，着力打造廉洁高效、风清气正的人民政府。

各位代表！宿迁加快发展的光荣使命，鼓舞我们砥砺奋进；全市人民的信任期盼，鞭策我们勇往直前。让我们紧密团结在以习近平同志为核心的党中央周围，在市委的坚强领导下，在市人大、市政协和社会各界的监督支持下，同心同德，开拓创新，以建设“强富美高”新宿迁的崭新业绩，迎接党的十九大胜利召开！
